# Loss of Primary Cilia Potentiates *BRAF/MAPK* Pathway Activation in Rhabdoid Colorectal Carcinoma: A Series of 21 Cases Showing Ciliary Rootlet CoiledCoil (*CROCC*) Alterations

**DOI:** 10.3390/genes14050984

**Published:** 2023-04-27

**Authors:** Andrea Remo, Federica Grillo, Luca Mastracci, Michele Simbolo, Matteo Fassan, Maria Paola Cecchini, Giuseppe Miscio, Antonio Sassano, Paola Parente, Alessandro Vanoli, Giovanna Sabella, Guido Giordano, Emanuele Damiano Urso, Luigi Cerulo, Aldo Scarpa, Francesco Fiorica, Massimo Pancione

**Affiliations:** 1 Pathology Unit, Services Department, ULSS9 “Scaligera”, 37122 Verona, Italy; 2 Anatomic Pathology, Department of Integrated Surgical and Diagnostic Sciences (DISC), University of Genoa and Ospedale Policlinico San Martino, 16132 Genoa, Italy; 3 Department of Diagnostic and Public Health, Section of Pathology, University of Verona, 37135 Verona, Italy; 4 Department of Medicine (DIMED), Surgical Pathology Unit, University of Padua, 35128 Padua, Italy; 5 Veneto Institute of Oncology, IOV-IRCCS, 35128 Padua, Italy; 6 Department of Neurosciences, Biomedicine and Movement Sciences, Anatomy and Histology Section, University of Verona, 37135 Verona, Italy; 7 Unit of Pathology, Fondazione IRCCS Casa Sollievo della Sofferenza, 71013 San Giovanni Rotondo, Italy; 8 Anatomic Pathology Unit, Department of Molecular Medicine, University of Pavia, 27100 Pavia, Italy; 9 1st Pathology Division, Department of Pathology and Laboratory Medicine, Fondazione IRCCS Istituto Nazionale dei Tumori, 20133 Milan, Italy; 10 U.O.C. Oncologia Medica, Ospedali Riuniti Azienda Ospedaliera Universitaria, 71100 Foggia, Italy; 11 Department of Surgical, Oncological and Gastroenterological Sciences, University of Padua, 35128 Padua, Italy; 12 Department of Sciences and Technologies, University of Sannio, 82100 Benevento, Italy; 13 Department of Diagnostics and Public Health, University and Hospital Trust of Verona, ARC-Net Research Center, 37135 Verona, Italy; 14 Radiotherapy Unit, Oncology Department, ULSS9 “Scaligera”, 37122 Verona, Italy

**Keywords:** rhabdoid colorectal tumors, rare cancers, Ciliary Rootlet Coiled-Coil (CROCC), *SMARCB1*

## Abstract

A rhabdoid colorectal tumor (RCT) is a rare cancer with aggressive clinical behavior. Recently, it has been recognized as a distinct disease entity, characterized by genetic alterations in the *SMARCB1* and Ciliary Rootlet Coiled-Coil (*CROCC*). We here investigate the genetic and immunophenotypic profiling of 21 RCTs using immunohistochemistry and next-generation sequencing. Mismatch repair-deficient phenotypes were identified in 60% of RCTs. Similarly, a large proportion of cancers exhibited the combined marker phenotype (CK7-/CK20-/CDX2-) not common to classical adenocarcinoma variants. More than 70% of cases displayed aberrant activation of the mitogen-activated protein kinase (MAPK) pathway with mutations prevalently in *BRAF* V600E. SMARCB1/INI1 expression was normal in a large majority of lesions. In contrast, ciliogenic markers including CROCC and γ-tubulin were globally altered in tumors. Notably, CROCC and γ-tubulin were observed to colocalize in large cilia found on cancer tissues but not in normal controls. Taken together, our findings indicate that primary ciliogenesis and MAPK pathway activation contribute to the aggressiveness of RCTs and, therefore, may constitute a novel therapeutic target.

## 1. Introduction

Patients with rare cancers account for as much as 24% of all cancer diagnoses. Commonly, patients with rare cancers have fewer treatment opportunities and are understudied at the level of genomic targets. A malignant rhabdoid tumor is a rare and extremely aggressive entity. The most frequent location of this tumor is the kidney with a prevalence in childhood. Adult forms are uncommon, and they are characterized by a poor prognosis. The extrarenal localization of these tumors has been described mainly in the central nervous system (called atypical teratoid/rhabdoid tumors), liver, soft tissues, colon, and rectum [1,2,3].

The localization in the colon and rectum is uncommon and, since 1990, fewer than 30 cases of rhabdoid colorectal tumors (RCTs) have been reported [4,5]. RCTs often arise in the right colon and in elderly patients [6,7]. Although the exact incidence and risk factors associated with rhabdoid carcinomas are unknown, Agaimy et al. reported that the most common site of this type of tumor in the gastrointestinal tract was the stomach, followed by the colon, small intestine, and esophagus [8]. The loss of the chromatin remodeling gene *SMARCB1* (INI1) due to inactivating mutation and/or deletion is characteristic of malignant rhabdoid tumors arising in childhood [6,7]. In contrast, the loss of *SMARCB1* is uncommon in RCTs, supporting the hypothesis that RCTs exhibit distinct molecular features from their pediatric counterpart [8,9,10]. As a consequence, the World Health Organization (WHO) classifies RCTs as carcinosarcomas, within the broader colorectal tumor family.

Over the last five years, new investigations on the molecular causes of RCTs identified key defects in the centrosome [9,10]. The centrosome is a biomolecular condensate composed of two microtubule-based, barrel-shaped centrioles, and a surrounding network of proteins named pericentriolar material (PCM) that is able to build and position the mitotic spindle and cilia in metazoans [11]. During cilia assembly, typically occurring at the quiescent G0 phase of the cell cycle (post-cytokinesis), basal bodies facilitate the formation of the ciliary rootlet, a fibrous structure composed mainly of rootletin also called CROCC (ciliary rootlet coiled-coil). Mutant *CROCC* has been found in a subset of RCTs [9,10,12]. In addition to its function at the base of the basal body, rootletin is also a core component of a proteinaceous centrosome linker, a poorly understood cohesion machinery that tethers the proximal ends of the two centrioles during the interphase [9,12,13].

Defects in the structure and function of primary cilia lead to a range of multifaceted disease phenotypes termed ciliopathies [14]. A number of key signaling pathways, including Hedgehog and Wnt pathways, are facilitated by primary cilia. This finding supports the hypothesis that primary cilia could have a role in cancer [15,16]. However, the way primary cilia regulate tumorigenesis seems to differ between tumor types and within tumor subtypes [15,16]. We therefore aimed to investigate the role of primary cilia in RCTs.

We here show that alterations in primary cilia and MAPK contribute to the aggressiveness of RCTs with dismal prognosis.

## 2. Materials and Methods

### 2.1. Patients

Twenty-one primary RCTs and matched normal, formalin-fixed paraffin-embedded (FFPE) samples were recruited from different medical institutions: (a) Konkuk University School of Medicine, Seoul, Korea; (b) Hospital Santariskiu Clinics, National Affiliate of Vilnius University, Vilnius, Lithuania; (c) Mater Salutis’ Hospital, ULSS9 “Scaligera” Legnago, Verona, Italy; (d) University of Genova, Italy ([9] and Ref therein).

Tumors with pleomorphic, giant, anaplastic, or undifferentiated histology were not included. The hematoxylin and eosin–stained (H&E) glass slides were independently reviewed by pathologists (A.R., F.G., L.M.) to confirm the diagnosis of RCT [9]. The twelve RCTs included in this study have been previously described [9]. The remaining cases (RCI-RCXII) have been identified and recruited for the present work. The tumors were staged using the conventional tumor, node, metastasis (TNM) staging system. No patient received chemotherapy or radiation therapy prior to surgery. Those patients who had a family history of intestinal dysfunction, CRC or who had taken non-steroidal anti-inflammatory drugs on a regular basis were not included. The diversity of the group’s ethnicity was not evaluated as more than 90% of the population was *Caucasian*. The overall survival (OS) time was available and defined as the time elapsed between the start of first-line chemotherapy and death. Those patients who were alive or lost in follow-up were censored at the last date they were known to be alive. Other clinical parameters, including biochemical and anthropometric variables, were not available. As treatment regimes after surgery were available for some but not all of the patients who participated, this information was not considered. The study was approved by the ethical committees according to the institutes’ ethical regulations on research conducted on human tissues. AOU Legnago-Verona: Ethics Committee Approval n. CA 2207 2016, September, 2016.

### 2.2. FFPE DNA Extraction

The rhabdoid component was identified by H&E staining. Manual macrodissection was used to isolate the rhabdoid component from FFPE blocks. DNA was extracted using Qiagen’s QIAamp DNA FFPE Tissue Kit according to the manufacturer’s instructions as reported [9,17].

### 2.3. Multipanel NGS Target Sequencing

A multigene panel was developed using AmpliSeq designer software v2.1. The panel included the following target genes: *CROCC*, *PTEN*, *FBXW7*, *MLH1*, *SMARCB1*, *KIT*, *MET*, *PDGFRA*, *PLCG1*, *PTPN11*, *ARAF*, *ATR*, and *DNMT3A*. PCR-amplified fragments from 20 ng of DNA were used to build an adequate library for deep sequencing in all cases. Emulsion PCR was performed with the Ion OneTouch OT2 System (Life Technologies, Carlsbad, CA, USA). A minimum coverage of 20× was obtained in all cases. The mean read length was 112 base pairs and a mean coverage of 6290× was achieved, with 94.1% target bases covered more than 100×. The quality of the obtained libraries was evaluated by the Agilent 2100 Bioanalyzer on-chip electrophoresis (Agilent, Santa Clara, CA, USA). Sequencing was run on the Ion Personal Genome Machine (Life Technologies) loaded with Ion 318 Chip v2. Data analysis, including alignment to the hg19 human reference genome and variant calling, which was performed using the Torrent Suite Software v.5.0 (Thermo fisher scientific, Whaltam, MA, USA). Filtered variants were annotated using a custom pipeline based on vcflib (https://github.com/ekg/vcflib, accessed on 17 February 2023), SnpSift, the Variant Effect Predictor (VEP) software, and NCBI RefSeq database. Additionally, alignments were visually verified with the Integrative Genomics Viewer (IGV) v2.3 to further confirm the presence of mutations identified by targeted sequencing.

### 2.4. Immunohistochemical Analysis

Immunohistochemical (IHC) analysis was performed on tumors and matched normal tissue sections as reported [9]. Four µm thick sections were deparaffined with BOND DEWAX Solution (Leica biosystems, Newcastle, UK), placed in graded alcohol solutions, washed, and pre-treated with the Epitope Retrieval Solution 2 (EDTA buffer pH 8.8) at 98 °C for 20 min. After the washing steps, peroxidase blocking was carried out for 5 min using the Bond Polymer Refine Detection Kit DS9800 (Leica biosystems, Newcastle, UK). Then, the sections were incubated with a rabbit primary anti-human CROCC rabbit polyclonal antibody (clone NBP1-80820, dilution 1:200, (ABCAM, Cambridge, UK)), or an anti-human γ-tubulin mouse monoclonal antibody (clone TU-30, dilution 1:100 (ABCAM, Cambridge, UK)) for 15 min, and a secondary antibody for 8 min, respectively. Subsequently, the sections were incubated with polymer (8 min), revealed with diaminobenzidene-chromogen (10 min), and stained with hematoxylin (10 min). In normal, non-pathological tissues, CROCC staining consists of up to two dot-like signals [9]. Consistently, we considered one or two signals per cell as quantitatively physiological. In contrast, abnormal staining was recorded as follows: (a) loss, less than 1 signal per cell; (b) amplified, more than 2 signals per cell. Two hundred cells in triplicate sections per sample were blindly scored by three authors (A.R., E.M., M.P.). Samples were also immunostained with the following markers: cytokeratin (CK)7, CDX2, CK20, SMARCB1/INI1, and mismatch repair (MMR) proteins (MLH1, PMS2), and scored as previously reported [9].

### 2.5. Dual Immunofluorescence Staining

A dual immunofluorescence method was employed to detect cilia at the level of single cells in tissue samples, as reported [9]. FFPE sections of 5 µm thickness were prepared and antigen retrieval was performed using the Universal HIER antigen retrieval reagent, 10× diluted 1:10 (ab208572, abcam), and heated at 97 °C using a decloaking chamber for 20 min. Slides were washed in washing buffer (PBS and 0.1% Tween 20 at 25 °C) followed by blocking buffer (5% normal goat serum, 0.1 M Tris-HCl, and 0.15 M NaCl, pH 7.6 at 25 °C) for 30 min at room temperature. Primary antibodies were diluted in blocking buffer and incubated at 4 °C overnight in a humidified chamber with anti-CROCC or anti-human γ-tubulin diluted 1:100. Slides were washed three times in washing buffer and incubated with secondary antibody and Dapi Staining Solution for DNA, diluted 1:10,000, for 30 min at room temperature. Secondary antibodies were: Goat Anti-Mouse IgG H&L (Alexa Fluor^®^ 647, red) (abcam, ab150115) and Goat Anti-Rabbit IgG H&L (Alexa Fluor^®^ 488, green) (ab150077), diluted 1:500. Slides were washed three times in washing buffer, mounted using Anti-Fade Fluorescence Mounting Medium—Aqueous, Fluoroshield (ab104135, abcam), and stored at −20 °C. Specimens were imaged using a Confocal Microscope (Nikon AXR system, 60× objective) equipped with a Ti2-E Inverted Microscope 25 mm FOV high-speed Resonant scanner and high-resolution Galvano scanner. High resolution images were acquired with NISElements C Software (N). The staining of cilia was considered abnormal if it showed a diameter greater than the diameter of signals present in normal epithelium within the same section.

### 2.6. High Resolution Melting (HRM) Analysis

Loss of heterozygosity (LOH) analysis was performed using fluorescent-labeled forward primers, followed by fragmental analysis detection on Rotor-gene Q 5plex HRM (Qiagen, Hilden, DE, USA), as reported [9]. Tumoral and matched normal DNA samples were analyzed for *CROCC* LOH using three markers: D1S3391, D1S1443 and D1S3669. LOH in SMARCB1 was investigated with the markers D22S301 and D22S345, as reported [9]. PCR conditions for the multiplex PCR consisted of: initial denaturation at 95 °C for 15 min, followed by 19 cycles of 95 °C 30 s, 55 °C 90 s, 72 °C 1 min, and a final extension of 72 °C 30 min. PCR reactions were performed in a 10 μL reaction mixture by using 50 ng template DNA, 0.2 mM dNTPs (Roche, Penzberg, DE, USA), 0.4 U FastStart Taq DNA Polymerase (Roche, Penzberg, DE, USA), 1× fluorescent dye LCGreen Plus (Idaho Technology, Salt Lake City, UT, USA), 2 mM MgCl_2_, and forward and reverse primers (0.5 mM each) for each gene segment. PCR conditions were optimized to temperatures between 52 °C and 64 °C for each segment. After 30 cycles of amplification, PCR products underwent an additional 1 min at 98 °C and then 5 min at 40 °C to promote heteroduplex formation. Each capillary was then transferred to the High-Resolution Melter instrument (HR-1) (Idaho Technology, Salt Lake City, UT, USA) for high-resolution melting and curve analysis. Samples were melted at 0.2 °C/s ramp rate. Melting profiles were analyzed with HR-1 software using fluorescence normalization, temperature shift and conversion to difference and derivative plots. To confirm the reliability of the HRM assay, selected paired non-neoplastic tissue samples showing differences or showing no difference in melting profiles were purified and then sequenced in both directions using the Big Dye Terminator 1.1 Cycle Sequencing kit (Applied Biosystems, Foster City, CA, USA). The sequencing reaction was performed on an automatic sequencer ABI PRISM 310 Genetic Analyzer and sequences were analyzed using BioEdit program.

### 2.7. Statistical Analysis

Patients were compared for survival outcomes, using both Kaplan-Meier survival curves. Differences in Kaplan-Meier curves were tested using the log-rank test. Genetic changes among subgroups were tested for statistical significance using the *t*-test (2-tailed). Statistical analyses were conducted by GeneSpring R/bioconductor v.12.5 and R based package and GraphPad Prism 5.

## 3. Results

### 3.1. Clinical Information and Follow-Up

A total of 21 primary RCTs diagnosed between 2007 and 2015 were included in this study.

The large majority of tumors (20/21; 95%) resulted as composites and only one (1/21; 5%) was diagnosed as a pure RCT, in which rhabdoid morphology is observed in virtually all tumor cells.

At diagnosis, the age of the patients ranged between 49 and 87 years (mean 71 years). The male-to-female ratio was 8:13 (38% versus 62%), respectively. The neoplasms were predominantly located in the right side of the colon (13/21; 62%), followed by left side (6/21; 28%) and rectum (2/21; 10%). The median tumor size was 6 cm (with a range of 3.5–10 cm). The most common tumor stage was pT3 (13/21; 62%%), followed by pT4 (8/21, 38%). Loco-regional lymph nodes were positive (N1, N2) in the majority of patients (14/21, 67%) and negative (N0) in a third of patients (7/21, 33%). Distant metastases (M1) were identified in 4 cases and localized to the liver. One case (RCIV) presented multiple liver and lung metastases. The available clinicopathological characteristics are reported in Table 1. The median overall survival of patients was 13.1 months (with a range of 1–43 months) (Table 1). Of the clinicopathological variables, only patients with lymph node involvement (N1–N2) had shorter overall survival than those without lymph node metastasis (Table 1).

### 3.2. *Immunophenotypic Profiling* of RCTs

Of the 21 tumors tested by IHC, 13 (62%) exhibited loss of nuclear expression of MMR proteins (MLH1/PMS2) and they were classified as deficient (MMR-d). The remaining 8 tumors (38%) exhibited intact expression of all mismatch repair proteins and they were classified as MMR proficient (MMR-p).

**Figure 1:**
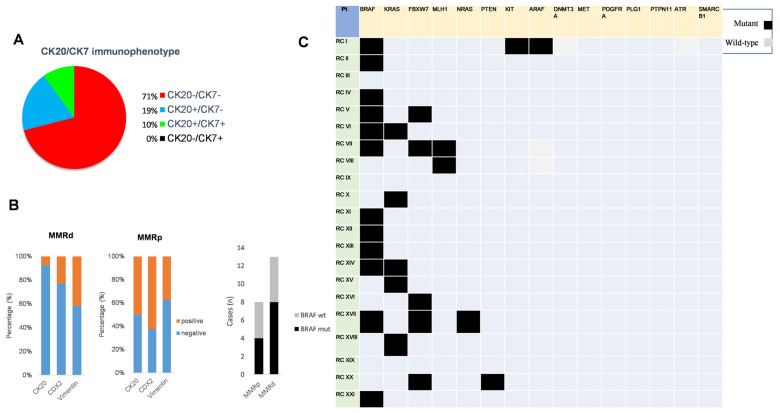
Immunophenotypic and genetic features of RCTs. (**A**) The combined CK20/CK7 immunostaining profiles in RCTs is shown by a pie chart; (**B**) Left: CK20, CDX2 and vimentin expression in mismatch repair-deficient (MMR-d) and mismatch repair-proficient (MMR-p) subgroups is shown by graphs. Right: *BRAF* mutants or wild-type tumors are related to MMR-d and MMR-p subgroups; (**C**) The recurrence of mutant (black squares) and wild-type (grey squares) in the indicated genes for each patient is shown by a panel.

Notably, CK20, a specific marker for classical colorectal carcinoma, was expressed in 5/21 (24%) cases and the remaining 16 tumors (76%) were negative. CK7 resulted as absent in the large majority of cases 18/21 (85.7%) and just one case was positive. Therefore, the combined evaluation of both markers revealed that the CK20−/CK7− pattern included the greatest proportion of cases 15/21 (71%), followed by CK20+/CK7− in 4/21 (19%), CK20+/CK7+ in 2/21 (10%), and CK20−/CK7+ in 0/21 (0%) (Figure 1A). CDX2 resulted as positive in 8/21 (38%) and negative in 13/21 (62%) tumors, respectively.

We then evaluated the expression of vimentin, a marker for the epithelial to mesenchymal transition (EMT). Vimentin-positive staining was found in 8/21 (38%) of RCTs, while the majority of cases were vimentin negative (13/21 (62%)) (Table 2).

Notably, negative expression of the epithelial markers CDX2 and CK20 was identified in more than 80% of MMR-d cancers compared to less than 50% of MMR-p cancers (Figure 1B). It is also of note that the IHC profile of vimentin was independent of MMR status (Figure 1B). The immunohistochemistry profiles of MMR, epithelial, and mesenchymal markers are shown in (Table 2).

### 3.3. Identification of Actionable Mutations in RCTs

The next-generation sequencing (NGS) analysis revealed the mutation *V600E* in *BRAF* as the most frequent with 12/21 (57%) (Figure 1C). Mutations in *BRAF* occurred regardless of MMR status (Figure 1B). Mutations in *KRAS* were identified in 5/21 (24%) cases, 2 of which co-occurred with *BRAF* mutations in the same lesion. The mutation profiles clearly indicated prevalent activating mutations in the *RAS-RAF-MAPK* pathway (15/21 (71.4%)) (Figure 1C). Recurrent mutations were also identified in the gene encoding for F-box and WD repeat domain containing 7 (*FBXW7*), with 5 of 21 (23.8%) cases (Figure 1C).

### 3.4. Defects in Primary Cilia and Rhabdoid Dedifferentiation

Although SMARCB1/INI1 is widely used as a marker of rhabdoid tumors, its relevance has not yet been comprehensively investigated in RCTs.

We thus analyzed SMARCB1/INI1 expression by IHC. Surprisingly, we found that only 3/21 cases (14%) exhibited loss of INI1 expression. In contrast, the large majority of RCTs (18/21 (86%)) exhibited an intense expression of SMARCB1/INI1 (Table 3).

**Figure 2:**
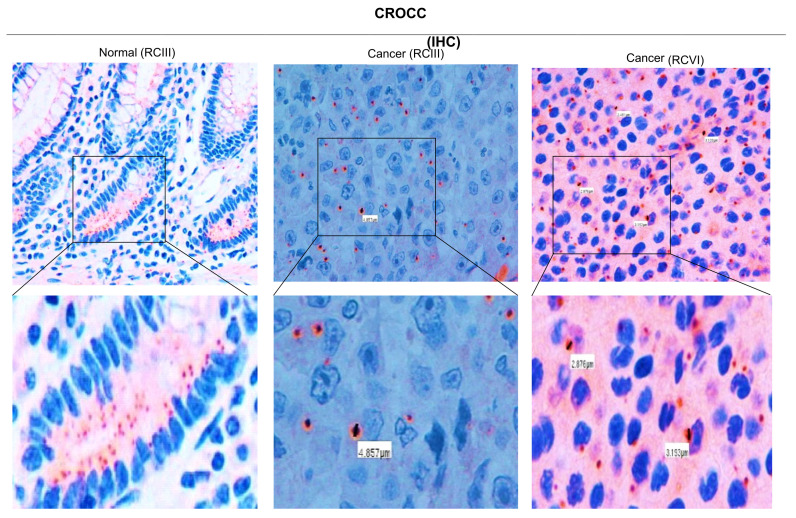
Immunohistochemistry of CROCC in RCT tissues. Left: CROCC IHC in colon normal tissues. Down panel: magnifications show that CROCC is expressed in normal mucosa. Right: CROCC immunohistochemistry in tumor tissues (RCIII and RCVI). Down panels: magnifications show that CROCC is absent in about 50% of tumor cells. Immunosignals foci near the nuclei appear as single and large foci. The size of CROCC-positive foci is shown by white rectangles.

**Figure 3:**
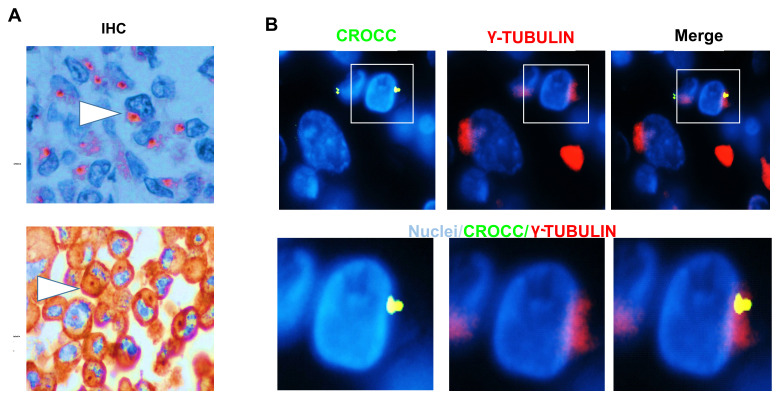
*CROCC* and γ-tubulin alterations in RCT tissues. (**A**) CROCC and γ-tubulin immunohistochemistry in RCT. Positive foci are shown by white arrows. (**B**) RCT tissue stained with γ-tubulin (red) and CROCC (green). Nuclei in blue. The magnification shows the colocalization of CROCC/γ-tubulin in abnormal cilia. Bar 10 μm.

As mutations in the ciliogenic gene CROCC have been reported in RCTs, we tested its expression by IHC. We found that the large majority of RCTs (19/21; 90%) exhibited no CROCC staining (Table 3). Notably, CROCC-negative coexisted with CROCC-positive cells, having large and apparently abnormal cilia compared to those detected in the matched normal mucosa (Figure 2). Single and abnormal large cilia were confirmed using IHC for γ-tubulin (Figure 3A). To verify the presence of abnormal cilia within tissues, we used immunofluorescence to investigate the colocalization of CROCC and γ-tubulin at cilia. We found that CROCC and γ-tubulin colocalized and appeared as large foci at ciliary basal bodies (Figure 3B).

We next investigated the DNA mutational profile and LOH at *SMARC1/INI1* and *CROCC* loci. The mutations and LOH in *CROCC* were identified in 5/21 (23.8%) and 12/21 (57%) cases, respectively. Of these, three cases exhibited the co-occurrence of missense mutation and LOH. In contrast, no mutations were identified in *SMARCB1/INI1*, while LOH was evidenced in 16/21 (76%) cases (Table 3). Notably, three cases with LOH in *SMARCB1/INI1* exhibited negative INI1 staining by IHC.

## 4. Discussion

RCTs represent a distinct entity that occurs in adult-elderly patients (31–87 years), mostly in the right colon without predisposition towards any one sex [9,18,19].

The diagnostic hallmark of an RCT is the rhabdoid morphology characterized by the presence of “large round neoplastic cells with glassy eosinophilic cytoplasm containing hyaline-like inclusion bodies, eccentric nuclei and macronucleoli” [8,9,20].

In this study, we analyzed a large series of 21 RCTs, 12 of which have already been described in the literature. The median overall survival time of patients was extremely short at “13.1 months” [8,9].

We included in the study RCTs with two pathological features: (a) mononuclear round cells with conspicuous eccentric macronucleoli and (b) binucleated large–medium sized cells with prominent nucleoli. Tumors with pleomorphic, giant, anaplastic, or undifferentiated colorectal carcinoma were excluded.

The lack of or negative expression of *SMARCB1*/INI1 was considered the predominant event in the development of pediatric rhabdoid tumors. However, the exact occurrence of *SMARCB1*/INI1 loss in RCTs remains unknown. We found that *SMARCB1*/INI1 expression was positive in the large majority of RCTs (85%), supporting the notion that RCTs are not genetically related to their pediatric counterparts [20].

A recent study on 3051 cases of classical CRC showed that *SMARCB1/INI1* is deleted in 14 cases (0.46%), confirming the previous data obtained by our group [7,8,9,21]. Therefore, the role of *SMARCB1/INI1* in the pathogenesis of RCTs may not be as relevant as initially thought.

The malfunctioning of the MMR repair pathway in RCTs is unknown. Unexpectedly, we found that more than half of RCTs are MMR-d cancers. In addition, the loss of expression of CDX2/CK20 was often associated with the MMR-d (MLH1/PMS2) subgroup [22]. The low prevalence of vimentin expression suggests that it is not a diagnostic marker of rhabdoid differentiation. Further studies are needed to understand whether the EMT process is involved in the rhabdoid pathogenesis.

The *BRAF* V600E mutation was identified in about 60% of RCTs. Interestingly, more than 70% of lesions displayed activating mutations in the MAPK pathway. These findings support the idea that RCTs prevalently arise and progress through serrated precursor lesions. However, mutations in *BRAF* were not correlated with MMR status. Thus, *BRAF* mutations and MMR, rather than causative events, could shape a permissive microenvironment for the progression to rhabdoid carcinomas. However, the precise molecular events need to be further investigated in future studies.

We recently found that the depletion of *CROCC* in *BRAF*-mutant colorectal cancer cells promotes the acquisition of rhabdoid features [8]. Primary cilia play an important role in the regulation of cell signaling pathways but have seldom been studied in rare cancer variants. Our study confirms the prominent role of centrosome/cilia in the development of rhabdoid features, expanding the spectrum of lesions at the *CROCC* locus that characterize such rare lesions.

Rhabdoid cells often exhibited single and aberrantly larger cilia as compared to normal tissues, suggesting an elevated level of genetic heterogeneity and chromosomal instability typical of rare cancer variants. By contrast, *CROCC* has been reported as significantly increased in classical CRC [9,23]. Therefore, our study suggests that RCTs and classical CRCs exhibit different abnormalities in primary cilia. Interestingly, our data indicate that the loss of primary cilia could potentiate *BRAF/MAPK* pathway activation, thereby explaining the rapid malignant progression and resistance to therapies of these neoplasms. Recent studies suggest that the primary cilium is a critical determinant in controlling tumor pathway switching. For example, in basal cell carcinoma, the primary cilium acts as an important lineage gatekeeper, preventing Hedgehog to Ras/MAPK pathway switching [24]. We propose that the duplication or division of the primary cilia could not occur under normal circumstances in RCTs, leading to marked defects in cellular signaling and microtubules dynamics. Thus, the close inverse association between mutations in the MAPK pathway and loss of primary cilia could be clinically exploitable in the development of selective therapies for patients with RCTs.

## 5. Conclusions

Taken together, our findings indicate that primary ciliogenesis and MAPK pathway activation contribute to the aggressiveness of RCTs and, therefore, may constitute a novel therapeutic target.

## Figures and Tables

**Table 1:** Clinicopathological features of RCTs enrolled in this study. n.a. = not available, OS = Overall Survival.

n° Case	Years	Center	Age (ys)	Gender	Site	Size (Max)	T	N	M	Type	OS (Months)	Status
RCT I	2009	Legnago	73	Female	Right colon	10	4	1b	none	composite	6	dead
RCT II	2009	Benevento	71	Male	Right colon	10	4	1a	none	pure	8	dead
RCT III	2009	Seoul	62	Male	Sigma	4.5	3	1a	none	composite	36	alive
RCT IV	2010	Seoul	83	Female	Rectum	6.5	4	2b	liver, lung	composite	1	dead
RCT V	2011	Vilnius	49	Male	Sigma	7	n.a.	2b	none	composite	7	dead
RCT VI	2007	Legnago	63	Male	Left colon	6	3	2a	none	composite	1	dead
RCT VII	2014	Legnago	71	Female	Right colon	8	4	2a	none	composite	8	dead
RCT VIII	2015	Legnago	66	Male	Sigma	8	4	0	n.a.	composite	n.a.	n.a.
RCT IX	2015	Legnago	85	Male	Anastomosis post-Bilroth	n.v.	4	0	n.a.	composite	1	dead
RCT X	2015	Genova	71	Female	Rectum	5	3	2b	none	composite	1	dead
RCT XI	2014	Genova	40	Female	Right colon	4	4a	2b	none	composite	18	dead
RCT XII	2014	Genova	72	Female	Right colon	5.2	3	0	none	composite	33	alive
RCT XIII	2015	Genova	87	Female	Right colon	7	3	0	none	composite	18	alive
RCT XIV	2015	Genova	78	Female	Trasversum colon	3.5	3	2b	liver	composite	13	dead
RCT XV	2013	Genova	65	Female	Right colon	3.5	3	0	none	composite	36	alive
RCT XVI	2014	Genova	81	Male	Right colon	7	3	1a	none	composite	7	dead
RCT XVII	2013	Genova	76	Female	Right colon	8.5	3	0	none	composite	43	alive
RCT XVIII	2014	Genova	73	Male	Right colon	5	3	2b	liver	composite	7	dead
RCT XIX	2015	Genova	81	Female	Right colon	7	3	1a	liver	composite	5	dead
RCT XX	2015	Genova	86	Female	Right colon	4	4b	0	none	composite	1	dead
RCT XXI	2015	Alaska	74	Female	Right colon	4.5	3	positive	none	composite	n.a.	dead

**Table 2:** Immunohistochemistry Profile of the RCTs.

n° Case	MMR	CK7	CK20	CDX-2	Vimentin
RCT I	MMRd (mlh1/PMS2)	neg.	neg	neg.	++
RCT II	MMRd (mlh1/PMS2)	neg.	neg	neg.	++
RCT III	MMRp	n.a.	++	++	neg.
RCT IV	MMRp	n.a.	neg	++	++
RCT V	MMRp	neg.	neg	neg.	++
RCT VI	MMRp	neg.	neg	neg.	++
RCT VII	MMRd (mlh1/PMS2)	neg.	neg	neg.	++
RCT VIII	MMRd (mlh1/PMS2)	neg.	neg	++	neg.
RCT IX	MMRd (mlh1/PMS2)	neg.	Neg. (10%+)	++	Focally +
RCT X	MMRp	neg.	++	++	neg
RCT XI	MMRp	++	++	++	neg
RCT XII	MMRd (mlh1/PMS2)	neg.	neg	neg	++
RCT XIII	MMRd (mlh1/PMS2)	neg.	neg	neg	neg
RCT XIV	MMRd (mlh1/PMS2)	neg.	++	++	neg
RCT XV	MMRp	neg.	++	++	neg
RCT XVI	MMRd (mlh1/PMS2)	neg.	neg	neg	neg
RCT XVII	MMRd (mlh1/PMS2)	neg.	neg	neg	neg
RCT XVIII	MMRp	neg.	neg	neg	neg
RCT XIX	MMRd (mlh1/PMS2)	neg.	neg	neg	neg
RCT XX	MMRd (mlh1/PMS2)	neg.	neg	neg.	neg
RCT XXI	MMRd (mlh1/PMS2)	neg.	neg	neg.	n.a.

**Table 3:** The DNA mutational profile and Loss of heterozigosity (LOH) at *SMARC1/INI1* and *CROCC* loci is correlated with immunohistochemistry (IHC). Results: n.a. = not available, neg. = negative.

n° Case	*CROCC* Gene	*CROCC* Loh	CROCC Ihc	*SMARCB1/INI1* Gene	*smarcb1/ini1* loh	INI1 ihc
RCT I	c481G>T; c.3705-2A>G	present	Loss (<1 signal/cell)	wild type	present	neg.
RCT II	c5654T>C	present	Loss (<1 signal/cell)	wild type	present	++
RCT III	wildtype	present	Loss (<1 signal/cell)	wild type	present	++
RCT IV	wildtype	present	Loss (<1 signal/cell)	wild type	present	++
RCT V	wildtype	present	Loss (<1 signal/cell)	wild type	present	++
RCT VI	wildtype	present	Loss (<1 signal/cell)	wild type	present	neg.
RCT VII	wildtype	present	Loss (<1 signal/cell)	wild type	intact	++
RCT VIII	wildtype	intact	Loss (<1 signal/cell)	wild type	intact	++
RCT IX	wildtype	intact	Loss (<1 signal/cell)	wild type	present	++
RCT X	wildtype	present	Loss (<1 signal/cell)	wild type	intact	++
RCT XI	Leu1159Phe	present	Loss (<1 signal/cell)	wild type	present	++
RCT XII	wildtype	intact	Loss (<1 signal/cell)	wild type	present	++
RCT XIII	wildtype	intact	Loss (<1 signal/cell)	wild type	intact	++
RCT XIV	wildtype	intact	Loss (<1 signal/cell)	wild type	present	++
RCT XV	wildtype	present	Loss (<1 signal/cell)	wild type	present	++
RCT XVI	Ala1510Thr	intact	Loss (<1 signal/cell)	wild type	present	++
RCT XVII	Ser1320Ile + Arg1659His	intact	Loss (<1 signal/cell)	wild type	present	++
RCT XVIII	wildtype	present	Loss (<1 signal/cell)	wild type	present	++
RCT XIX	wildtype	intact	Loss (<1 signal/cell)	wild type	present	++
RCT XX	wildtype	present	Loss (<1 signal/cell)	wild type	present	++
RCT XXI	wildtype	n.a.	Loss (<1 signal/cell)	wild type	n.a.	neg.
